# Study protocol: International joint research project ‘climate change resilience of Indigenous socioecological systemsʼ (RISE)

**DOI:** 10.1371/journal.pone.0271792

**Published:** 2022-07-21

**Authors:** Jorge García Molinos, Tuyara Gavrilyeva, Pattamaporn Joompa, Daiju Narita, Sinee Chotiboriboon, Varvara Parilova, Solot Sirisai, Innokentiy Okhlopkov, Zhixin Zhang, Natalia Yakovleva, Prapa Kongpunya, Sueppong Gowachirapant, Viacheslav Gabyshev, Wantanee Kriengsinyos

**Affiliations:** 1 Arctic Research Center, Hokkaido University, Sapporo, Japan; 2 Institute of Engineering and Technology, North-Eastern Federal University, Yakutsk, Russian Federation; 3 Department of Regional Economic and Social Studies, Federal Research Centre «Yakutian Scientific Center» of the Siberian Branch of the Russian Academy of Sciences, Yakutsk, Russian Federation; 4 Institute of Nutrition, Mahidol University, Nakhon Pathom, Thailand; 5 Graduate School of Arts and Sciences, The University of Tokyo, Tokyo, Japan; 6 Institute of Finances and Economics, North-Eastern Federal University, Yakutsk, Russian Federation; 7 Emeritus Researcher Faculty of Liberal Arts, Mahidol University, Nakhon Pathom, Thailand; 8 Institute for Biological Problems of Cryolithozone of Siberian Branch of the Russian Academy of Sciences, Yakutsk, Russian Federation; 9 KEDGE Business School, Paris, France; International Maize and Wheat Improvement Centre: Centro Internacional de Mejoramiento de Maiz y Trigo, MEXICO

## Abstract

**Background:**

Anthropogenic changes in the environment are increasingly threatening the sustainability of socioecological systems on a global scale. As stewards of the natural capital of over a quarter of the world’s surface area, Indigenous Peoples (IPs), are at the frontline of these changes. Indigenous socioecological systems (ISES) are particularly exposed and sensitive to exogenous changes because of the intimate bounds of IPs with nature. Traditional food systems (TFS) represent one of the most prominent components of ISES, providing not only diverse and nutritious food but also critical socioeconomic, cultural, and spiritual assets. However, a proper understanding of how future climate change may compromise TFS through alterations of related human-nature interactions is still lacking. Climate change resilience of indigenous socioecological systems (RISE) is a new joint international project that aims to fill this gap in knowledge.

**Methods and design:**

RISE will use a comparative case study approach coupling on-site socioeconomic, nutritional, and ecological surveys of the target ISES of Sakha (Republic of Sakha, Russian Federation) and Karen (Kanchanaburi, Thailand) people with statistical models projecting future changes in the distribution and composition of traditional food species under contrasting climate change scenarios. The results presented as alternative narratives of future climate change impacts on TFS will be integrated into a risk assessment framework to explore potential vulnerabilities of ISES operating through altered TFS, and possible adaptation options through stakeholder consultation so that lessons learned can be applied in practice.

**Discussion:**

By undertaking a comprehensive analysis of the socioeconomic and nutritional contributions of TFS toward the sustainability of ISES and projecting future changes under alternative climate change scenarios, RISE is strategically designed to deliver novel and robust science that will contribute towards the integration of Indigenous issues within climate change and sustainable agendas while generating a forum for discussion among Indigenous communities and relevant stakeholders. Its goal is to promote positive co-management and regional development through sustainability and climate change adaptation.

## Introduction

Indigenous Peoples (IPs) occupy over a quarter of the world’s surface area, encompassing approximately 40% of terrestrial protected areas and intact ecosystems that safeguard 80% of Earth’s biodiversity and 90% of its cultural diversity [1, 2]. The UN Declaration on the Rights of Indigenous Peoples recognises ’the right to the lands, territories and resources which [IPs] have traditionally owned, occupied or otherwise used or acquired.’ Within this context, food sovereignty and the sustainability of Indigenous traditional food systems are emergent issues within the sustainable development agenda, as recognised by the United Nations Permanent Forum of Indigenous Peoples’ Issues. The traditional food systems (TFS) of Indigenous Peoples can be broadly defined as ’all food within a particular culture available from local natural resources and culturally accepted’ [3]. Here, we follow this definition to refer to systems of hunting, fishing, and gathering that complement those of farming and animal breeding to provide a subsistence base from available natural resources. Notwithstanding the growing influence of external factors (e.g., industrialisation and access to technology), TFS are eminently knowledge-based systems rooted in the traditions, culture, and beliefs of IPs and the specific geographical settings of their ancestral territories. Although the diversity of Indigenous food varies regionally and seasonally, it is typically very high [4]. Even in harsh, extreme environments such as the Arctic, TFS can easily comprise over a hundred species [4, 5].

Food systems represent prominent socioecological systems that connect humans to the environment at multiple levels [6, 7]. This is particularly the case for Indigenous TFS because the kincentric view of human-nature relationships of IPs places themselves and nature as part of an extended ecological family of common ancestry and origins [8, 9]. For the purpose of our project, we focus on their socioeconomic and nutritional dimensions encompassing related local practices of processing and storage, consumption, marketing and trade undertaken at the household level within the community. These in turn are linked to well-being and health status through the influence of TFS on food security and nutrition.

Many historical and present-day factors contribute to the vulnerability of traditional Indigenous modes of livelihood, including social inequality, poverty, cultural assimilation, sovereignty issues, land appropriation, and the overexploitation of resources by external agents [1]. However, the inextricable link between IPs and nature makes TFS particularly exposed to climate change and environmental impacts [4, 6], which interact with other existing drivers to exacerbate vulnerability by increasing susceptibility to harm and hindering the capacity to cope and adapt [10]. Climate change can directly disrupt TFS by hindering Indigenous subsistence practices and access to food resources through alterations in the physical environment, and by altering the availability of food species through impacts on their distribution, abundance, and phenology [11]. For example, changes in sea ice conditions associated with climate warming are making access to marine mammal hunting grounds increasingly difficult and expensive for native coastal communities of northern and western Alaska while impacting the behaviour, health, abundance, and distribution of sea mammals [12]. These changes feed into pre-existing socioecological vulnerabilities generated by non-climatic drivers operating at multiple timescales. For example, the capacity of individual hunters and households to cope with the increasing costs of hunting under variable ice and snow regimes depends on their personal situation and financial resources at the moment those changes are experienced [13]. On the other hand, historical sociopolitical (e.g., colonialism, land usurpation, resettlement) and sociocultural (e.g., assimilation policies) transformations provide the background against which current drivers of vulnerability operate today. For example, the resettlement of 19 Inuit settlements in the Frobisher Bay region (Nunavut) into Iqaluit during the second half of the twentieth century resulted in the concentration of an increased population in a much reduced harvesting area, leading to increasing hunting pressure and land-use conflicts that are further exacerbated by recent changes in ice and climatic conditions [13].

Disrupted Indigenous TFS are characterized by increased food insecurity and inequalities, loss of traditional identity, westernization of livelihoods, and nutritional problems [6, 14]. The degree of vulnerability to these factors depends on their adaptation capacity and sensitivity, which in turn is conditioned by the degree of socioeconomic and nutritional reliance on traditional foods [7]. Mixed economies characterise the households of many Indigenous communities today, reflecting an increasing need for cash to access commodities and support traditional activities [15]. Changes in traditional food consumption patterns toward marketed or processed foods are increasingly common, especially among the younger population [16]. This process of rapid modernisation and westernisation of Indigenous lifestyles and livelihoods, including dietary shifts and altered energy consumption patterns (i.e., nutrition transition), has led to an increasing prevalence of chronic diseases, malnutrition, and mental health disorders [17, 18]. On the other hand, together with job opportunities, having access to locally available fish and game and a sense of local control remain key factors associated with IPs’ well-being and willingness to remain in Indigenous communities [19, 20]. Traditional foods chiefly contribute to dietary health and nutritional security [3, 21], more active lifestyles, cultural preservation, and, via food diversification, can increase adaptation and resilience [22], particularly to climate change, in Indigenous communities.

Understanding the complex relationships between climate change and TFS in the context of socioeconomic well-being and nutritional security of dependent Indigenous communities is crucial for informing climate change adaptation strategies and promoting the long-term sustainability of Indigenous socioecological systems in line with international agendas, such as the 2015 Paris Agreement on climate change and the UN’s 2030 Sustainable Development Goals. The sustainability of traditional foods and the scope for adaptation in Indigenous communities to the impacts of climate change and other environmental changes are conferred by their deep knowledge of nature and their ancestral bonds to it, including their connections and reliance on traditional foods (i.e., their traditional ecological knowledge). Recognition and incorporation of this knowledge are crucial in climate change adaptation [23], enabling IPs and other relevant stakeholders to assess the impacts of future climate and environmental changes and identify potential adaptation strategies.

**Fig 1:**
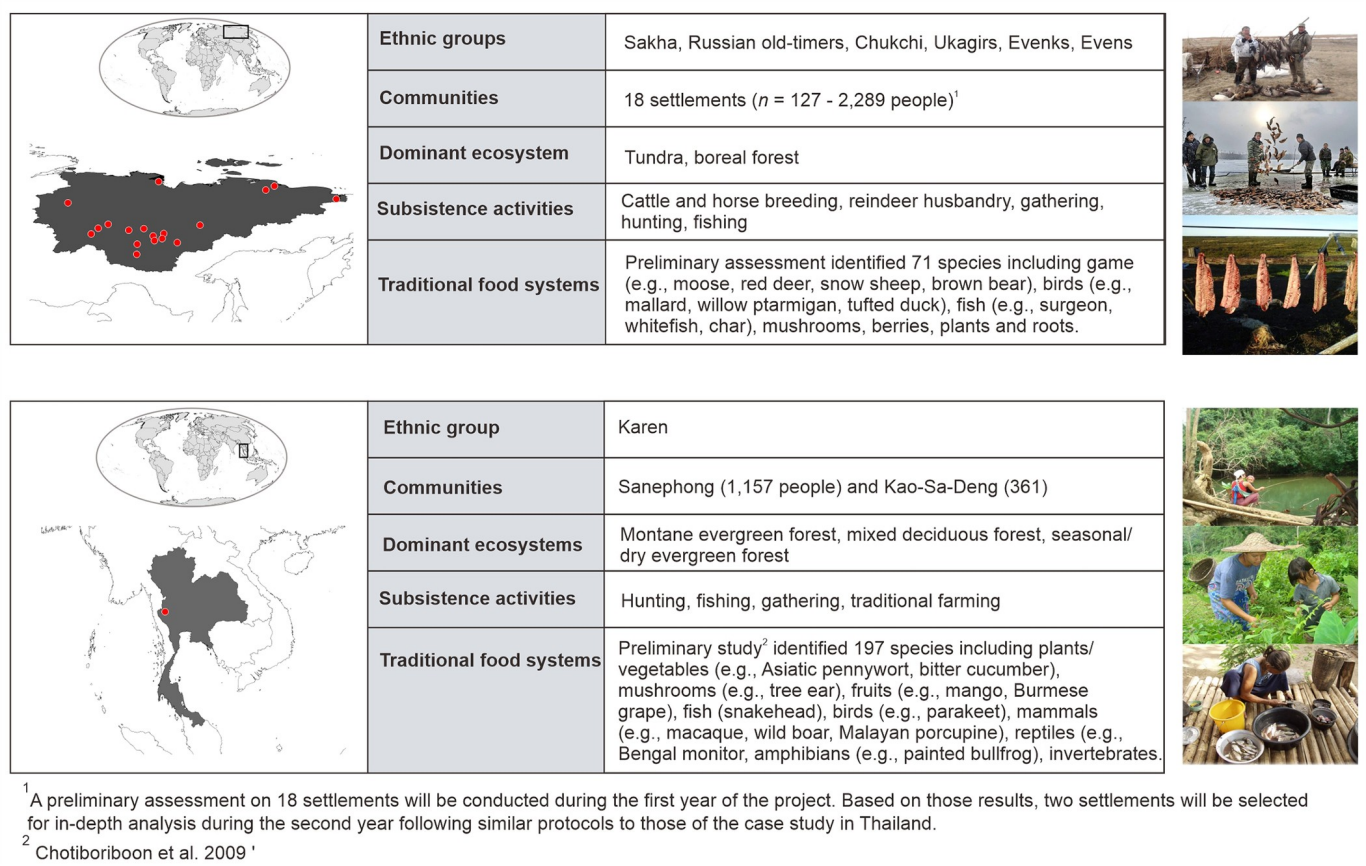
Location of the case study Indigenous socioecological systems and the main characteristics of their associated traditional food systems. The Karen people constitute the largest Indigenous cultural minority of Thailand, while the Sakha people encompasses 18 different cultural groups living in the Republic of Sakha in the Russian Far East. All photographs taken by the author team. World and individual country maps reprinted from the geoBoundaries data set v. 4.0. [27] under a CC BY license, with permission from geoBoundaries, original copyright 2020.

RISE aims to bridge this gap by using a novel, transdisciplinary analytical framework in a comparative case study context using contrasting socioecological systems of Indigenous communities from the tropics (Karen people in Thailand) and the Arctic (several IP groups from the Sakha Republic in the Russian Far East) (Fig 1, S1 Appendix). The Arctic and tropics are home to a large proportion of the world’s IPs and face some of the fastest and most intense environmental impacts resulting from the combined effect of multiple drivers, such as climate change, overexploitation of natural resources, land use change, and pollution originating from external, non-indigenous actors [24–26]. Based on a unified protocol for collecting and processing data during parallel field surveys with subsequent interdisciplinary analyses (see Materials and Methods), our project aims to

- Assess the contribution of traditional food systems as key socioecological systems connecting Indigenous communities to their environment and natural resources, toward their livelihoods and nutrition, identifying existing vulnerabilities and dependencies, and documenting adaptation responses to previous climatic and environmental changes experienced by the communities.
- Project future changes in the distribution and composition of local food species supporting TFS under alternative narratives of future climate change.
- Assess the risks to the sustainability of TFS posed by the combination of projected future changes in the composition and availability of food species (exposure) and the current dependency of the Indigenous communities on those species (vulnerability) evaluated from the information gathered in (i), as well as Indigenous traditional environmental knowledge.
- Use these risk scenarios as future narratives to promote a forum for discussion between Indigenous communities, local and regional authorities, and other relevant stakeholders on issues related to natural resource management, climate change adaptation, and food security. This will help to identify future research priorities and contribute to regional development by building the resilience of these communities against the plausible impacts of future climate and environmental change on their traditional food systems.

## Materials and methods

RISE was started in April 2021 and will run until March 2024. Our multidisciplinary research group is comprised of three national teams from Japan, Russia, and Thailand, each with complementary expertise in areas such as climate change ecology, environmental economics, social studies, anthropology, regional development, and nutrition science. Importantly, our teams have an established record of research collaboration with the Indigenous communities that is the focus of this project [28–31].

**Fig 2:**
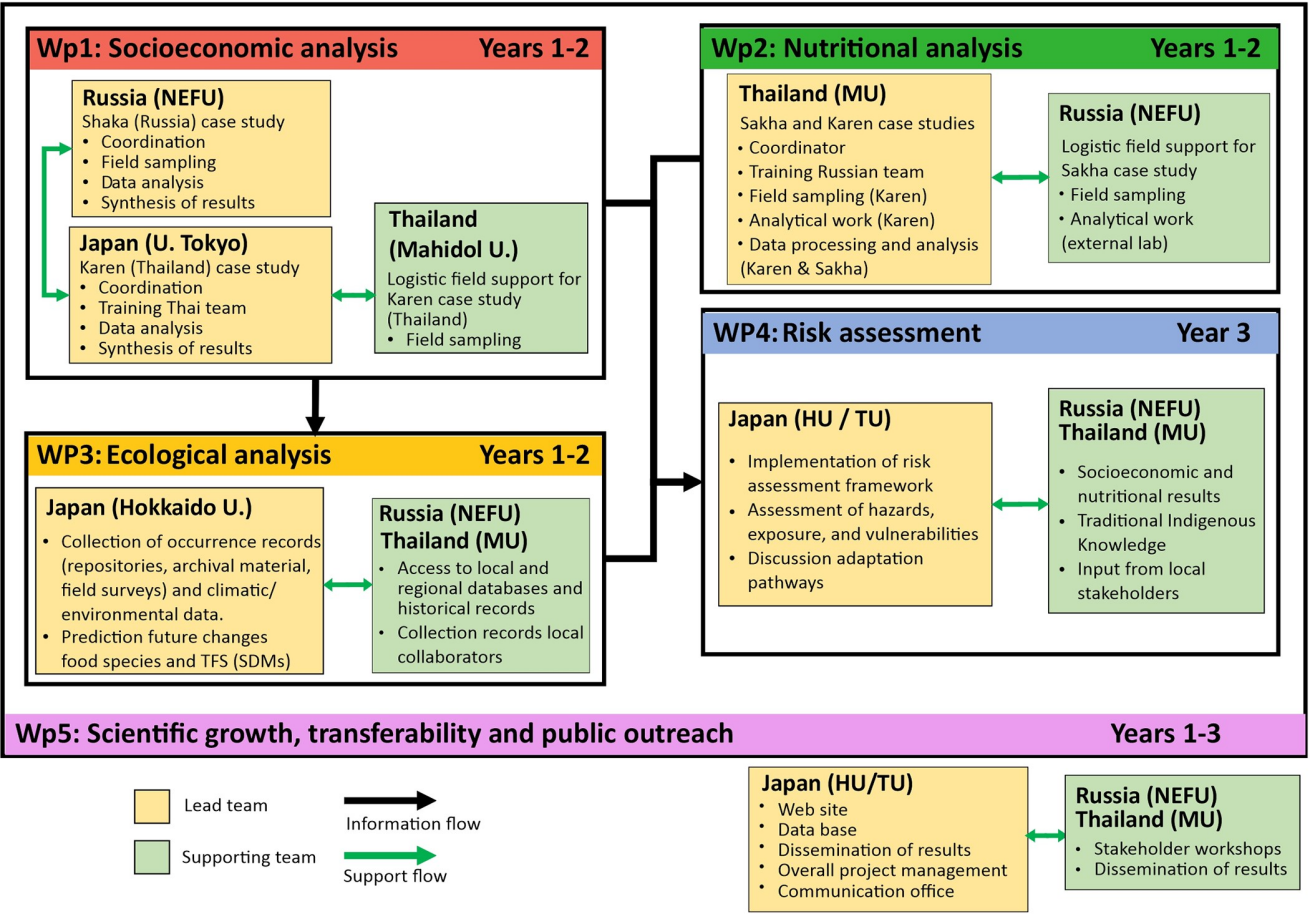
Flowchart for the project. Flowchart showing the different work packages (WP), their main goals, and relationships within the overall framework of the project.

RISE is structured into several work packages (WPs), each with a set of clearly defined complementary goals as described below (Fig 2). Field work (WPs 1 & 2) will be conducted during the first two years of the project in parallel in both case studies. Household questionnaires, focus group discussions, food frequency questionnaires, and dietary recalls will be combined to quantify the contribution of traditional food to the socioeconomic and nutritional status of the Indigenous communities. They will also be used to recollect community perceptions of local environmental and climatic changes, related observed impacts on traditional food species, and identify existing vulnerabilities and adaptation strategies toward these changes that have been taken by the communities. Notwithstanding the regional differences between the study sites (such as geographical, population, administrative, and logistical), the project aims to follow comparable protocols for fieldwork, case study research, sampling, and surveys, all of which are described in detail in the following sections.

Based on the information collected from the field surveys, a representative number of species will be selected to project the impacts of future climate change changes on TFS using an ensemble of species distribution models during the second year of the project (WP3). These are ecological models built from species occurrence data (response variables) and environmental predictors, the purpose of which is to predict the habitat suitability and distribution range of a species under current and future environmental conditions. The results from this modelling exercise will therefore provide potential changes in the distribution and composition of food species supporting the local TFS in the study communities under alternative emission scenarios.

The last year of the project will bring together Indigenous traditional environmental knowledge and the information generated from WPs 1 and 2 (informing on vulnerability of Indigenous TFS) and WP3 (informing on exposure of TFS). This will be used to analyse the relative risk as the potential for adverse effects that changes in traditional food systems generated by future changes in local climatic conditions may have on dependent Indigenous communities (WP4). The information, presented as alternative future narratives under different global circulation models (GCMs) and emission scenarios, will be used to identify potential future vulnerabilities and adaptation options with input from group discussions with the direct participation of Indigenous and other local stakeholders.

Finally, WP5 is responsible for the overall management of the project between the participating international teams and our Indigenous partners, as well as communication and outreach activities. RISE follows the recommended research practice [32, 33] by regarding local Indigenous People as full partners in research to ensure adequate joint development and benefit-sharing of the project according to their needs and objectives (see section ’Indigenous cultural and intellectual property (ICIP) rights and benefit-sharing’).

Ethical considerations and the safeguarding of the rights of the members from the Indigenous communities who will collaborate with us are a priority of our research project, which we will strive to maintain from the initial stages of the project to the execution of field work, data collection and analysis, generation and publication of research results, and subsequent record keeping. All procedures will be conducted to ensure proper treatment of the information and engage with the community in an inclusive, transparent way to develop trust, promote integrity of research, create mutual trust, and guard against misconduct and impropriety in their organisations or institutions. Above all, the active and voluntary participation of members from our study communities in our research project is designed to make the research as inclusive and useful as possible. It will be conducted with respect to their wisdom, customs, and livelihoods (see section ’Indigenous cultural and intellectual property [ICIP] rights and benefit-sharing’).

### Socioeconomic analysis (WP1)

The purpose of this package is to characterise household conditions in our study communities through the collection of economic data and traditional food use. To this end, as described below, we will conduct systematic household surveys for which we will develop and utilise questionnaires similar to those of national surveys in the target countries. Depending on the conditions of the localities, such as population and literacy, the survey may target all village households or a representative sample of village households. These quantitative surveys were supplemented by qualitative focus group discussions, in-depth interviews, and other ancillary sources of information (see below).

We will pay special attention to estimating the associated costs and time spent on the gathering and processing of traditional food by household, the use of and seasonal access to traditional food throughout the year, and existing dependencies on marketed goods and wage employment (i.e., the cost of modern life). Adopting the framework of the almost ideal demand system (AIDS) [34], we perform statistical regression analyses to determine the relationship between household economic conditions (expenditure, income, etc.) and other household characteristics and quantitatively estimate the elasticities of food availability conditions on the household economy (i.e., the household production function). Ethical considerations associated with each type of survey will be handled properly, as described below, and managed based on the procedures formulated in the IRB-approved protocols. Socioeconomic work comprises four levels of data collection.

1. Systematic household surveys will be conducted to obtain information about the socioeconomic characteristics of the households, such as their composition, educational and employment status, economic conditions (e.g., income, expenditure, assets), demography, and an inventory of food sources by household, including their type (natural or marketed) and consumption patterns (frequency and quantity). Key participants will be the family head or other members who play major roles in procuring and managing food in the household. The survey is based on official household socioeconomic surveys adapted to the requirements of our project (i.e., focus on traditional food systems) and case studies. We will adopt research ethical standards for qualitative one-to-one interviews [35, 36]. All participants will be adequately informed about the study purpose and content prior to the interview and asked to provide their free, prior, and informed consent (see section ‘Indigenous cultural and intellectual property [ICIP] rights and benefit-sharing’). To respect their right to reconsider their participation, participants will be informed before the start of the interview that they can withdraw their consent at any time during the course of the interview. To reduce the risk of unintended harm to the respondent, all participants will be informed before the interview that they can freely refuse to answer any question they feel uneasy or hesitant to respond to. The fact that local collaborators from the communities, previously trained by our research team, will help conduct the interviews, and that several members of our research team have worked previously in related research with these communities, provides a level of familiarity and trust that should facilitate communication on these sensitive issues, reducing the risk of unanticipated harm. All information and data collected by the survey will be treated and analysed anonymously.
  For the Karen case study, an all-household survey at each village will be conducted, contingent on the participation rate (initial contacts with community leaders suggest that the response rate will be high). Surveys will be repeated once a year for the first two years of the project to cover seasonal effects (winter 2021 for the dry season and spring 2022 for the wet season).
  In the Sakha case study, given the much larger scale of the study region and higher number of communities involved (Fig 1), a two-stage approach is to be followed instead. Eighteen settlements in each of the five geographical zones of Yakutia have been selected for the first stage (Fig 1). During the first year of the project, all communities will be visited once and anonymous surveys will be conducted for a representative number of households. The rural population of Yakutia of working age and older was selected as the pool of population to calculate the sample size because these people are most dependent on traditional food systems and are responsible for food decisions in their households. The total (i.e., across settlements) sample size for the surveys is calculated according to the Slovin formula [37]:
$$n=\frac{N}{\left(1+N*e^{2}\right)}$$ (1)
  Where *n* and *N* are the sample and total population sizes, respectively, and *e* is the level of precision (0.05 in this study). For the total registered population of 12,321 people of working age and older across all eighteen settlements (S1 Appendix), the resulting sample size is 387 people, which was finally rounded up to 400 participants. To maintain a balanced distribution of the sample size among settlements, both geographically and demographically, given the vast extension of the study area and size differences of the rural settlements, the total sample size was then divided among the 18 settlements according to the five recognised economic zones of Yakutia (i.e., Arctic and southern, central, eastern, and western Yakutia), and proportional to the population size of working age and older of each rural settlement (see S1 Appendix). Based on the results from the first-year survey, an all-household survey, similar to that conducted for the Karen case study in Thailand, will be conducted in two of the communities selected based on the degree of their dependence on traditional food systems. The two communities, contrasting in terms of their geographic and cultural-social characteristics, and the food and harvesting activities will be selected for this second stage of the research. The research methods and objectives match those of the Karen case study.
2. Focus group discussions (*n* = 12–18 with six participants each) and in-depth interviews (*n* = 30) were then used to understand the perceived effects of ongoing climate change on socioecological systems, the integrity of natural food species, and the adaptation capacity of Indigenous communities. The questions will focus on the social and cultural relations and obligations of Indigenous communities, including rituals, beliefs, and cultural traits, as well as patterns of use of natural resources in relation to the status quo of the ecological system and the integrity of natural/traditional food species according to ecosystem types. Questions will also address changes in socioecological systems over the last decade perceived by the community, particularly in relation to climate change, and any adaptation response made by the community to those changes. Similar to the household surveys, data will be collected for the first two years of the project to cover seasonal patterns.
  Although seasonal dynamics are better captured by multi-year longitudinal studies [13], we are limited by the duration of the project and available resources. Nonetheless, we will use the information collected in the interviews and focus-group discussions to detect whether the two years of field sampling are perceived as unusual by the locals and use historical records from nearby weather stations to check if they sit climatologically outside the regional historical baseline.
  Key participants in the interviews and group discussions will be community leaders and local residents who play roles in producing, hunting, gathering, cooking, and consuming natural foods according to each socioecological system. In order to gain a longer-term, historical (life-time) perspective of these changes, their drivers, and implications, a representative number of elders will also be included as the living memory of the community. Discussion sessions will be tape-recorded and will include taking notes and drawing pictures to communicate efficiently with the group participants. Focus group data, including all interviews, observation notes, and images or visual data, will then be transcribed.
  We will apply the triangulation method and follow the analytical framework proposed by Creswell and Creswell [38] for the sequential organisation and preparation of the data, data codification, and generation and representation of the description and themes. Given the distinctive ethical challenges of the focus group methodology relative to those of one-to-one interviews in terms of consent, confidentiality, and anonymity, as well as the risk of harm [39], we will follow specific recommended strategies to address and minimise these issues. First, to ensure their active consent during the consent process, we will read aloud and distribute among the participants an information sheet [40] to help participants create appropriate expectations by providing sufficient, clear information about the purpose of the focus group, examples of the types of questions that will be discussed, as well as clarification of what will be done with the data to be collected, and the steps to be taken to preserve confidentiality and/or anonymity.
  During the preliminary briefing session prior to starting the discussion, the moderator will then discuss and agree with the participants on a set of ground rules emphasising the public nature of a focus group and the need for confidentiality and anonymity, as well as provide guidance on identified subjects that the participants might feel as sensitive (e.g., specific information on the areas for collection of wild food species within the natural reserve). At this point, participants will be given the opportunity to withdraw their decision to participate in the group discussion if they wish to do so before the start of the group discussion. During the discussion, the moderator will strive to ensure that all members of the group have the opportunity to willingly contribute to the discussion, while remaining vigilant for signs of distress, breaches of confidentiality, or over-disclosure by the participants.
  Finally, a debriefing session will be provided after the focus group discussion, where the moderator reminds participants about the need for confidentiality and anonymity and gives an opportunity to address any remaining concerns or issues. To this end, the moderator will remain in the room for a while, as well as making himself or herself available for individual contact.
3. A survey of all food stores in each community will be conducted during the first two years of the project to produce a seasonal inventory of the type, quantity, frequency, and price of marketed food that is accessible to each community. Some local residents do not gather or hunt food species by themselves but still have access to seasonal food species through community food stores and mobile grocery stores (e.g., bicycles and motorcycles). Therefore, identifying the types of food available from these stores and their importance within the study communities is important for our study.

A search for ancillary demographic and socioeconomic data on the case study communities from official reports and archival material produced by regional and national government bodies, as well as published and unpublished research will be conducted during the first year of the project.

Both of the Karen community objects of study in RISE are matriarchal, with egalitarian decision-making based on the principles of mutual care, collaboration, consensus, and cosmological respect [41]. The decision process relies on both official and natural community leaders, who can be male or female, old or young, working or non-working members of the community, and who participate in the formation of community norms. Vulnerable or marginalised members are uncommon in these communities and refer chiefly to women, particularly single mothers, and the young who recently moved into the community, the disabled, and those who cannot provide themselves and their families with food and necessities, such as those with chronic ailments.

In the case of the Sakha case study, specific considerations related to social structure and relationships will be considered after the selection of the two study settlements based on the results of the first-stage survey that covers 18 settlements, including multiple ethnic minority groups across Yakutia. Traditional social structures in these communities include both patrilineal (e.g., Even) and matrilineal (e.g., Yukaghir) clans, although women often command relatively high positions in family and social life, even in patriarchal structures [42]. Overall, the situation of the dominant Turkic-speaking Sakha concentrated around the central and urban areas of the Sakha Republic is in clear contrast with the remote and isolated communities of small minority groups in its northern regions. The remoteness of these communities, with all the accompanying socioeconomic factors such as high unemployment rates, low living standards, and unreliable or non-existent communication, implies a high risk of marginalisation for vulnerable groups [43]. Common marginalised members include people with extremely low incomes and no relatives as well as alcoholics and people without a steady source of income. Immobile disabled, pensioners living alone, and single-parent families also have a higher risk of poverty.

To take the challenges faced by marginalised persons into consideration, we will use snowball sampling techniques to identify a representative number of vulnerable and/or marginalised members of our study communities. Identified members will then be asked to participate, where consent can be obtained, in the in-depth interviews and focus-group interviews. To do so, we will follow the recommended ethical considerations to design more inclusive processes [44] related to (1) ground rules (e.g., assuring and ensuring confidentiality in the interviews, ensuring equal right to speak, and clearly defining how community members’ views and ideas can be communicated in the focus groups), (2) space (e.g., selecting settings that make vulnerable participants feel comfortable), (3) facilitation (e.g., interviews conducted by our local collaborators who are regular members of the community), (4) documentation (e.g., outputs made available to participants in their local language), and (5) synthesis (e.g., ensuring results and research priorities are shared with the marginalised groups to give them a final say as a way to share power and allow their voices to be heard). They will also be invited to join project workshops and community meetings arranged with sufficient space and time for them to share their perspectives, problems, and needs with other community members and local stakeholders to jointly develop responses and solutions for capacity building and adjustment to cope with existing problems, ensuring that they have a representation and voice in our project. In doing so, we will follow a stepped approach [44], where small groups comprising vulnerable members with similar characteristics meet first in a safe space where they can express themselves and share their ideas comfortably before progressing into larger group discussions.

### Nutritional analysis (WP2)

This package focuses on understanding the role and importance of traditional foods in Indigenous diet and nutrition. Food composition analysis will be used to profile the nutritional value of representative Indigenous foods, locally available and consumed, relative to commercially available foods. Nutritional assessment will then be used to characterise the nutritional status and food consumption patterns of the IPs in each study community. The importance of traditional food in the diet of IPs will be considered from its seasonally adapted nutrient profile and consumption relative to their nutritional status [45].

Samples of raw Indigenous food products for food composition analysis will be collected from each study community following selection criteria based on their representability and availability, as well as their contribution to the dietary pattern of the local people in terms of consumption amount and frequency. Twenty samples of raw food products will be collected during the first two years of the project to capture seasonal effects (10 samples each during the 2021 dry season and the 2022 wet season), and 20–40 samples during the second year only for the Sakha case study during summer and winter (seasonality). The list of nutrient-dense foods to be sampled for analysis will be decided upon through discussions with representative household members who play a prominent role in gathering or cooking. The food list will include protein-rich foods (e.g., legumes) and foods important for their vitamin and mineral content (e.g., vegetables and fruits).

Food samples will be collected, prepared, and stored at -20°C for transportation to the analytical laboratory following standard procedures. Proximate composition analysis of foods, including moisture, protein, fat, dietary fibre, ash, and carbohydrate (by difference), will be determined according to the standard analytical procedures detailed by the Association of Official Analytical Chemists [46]. The food energy content per 100 g of the analysed foods will be estimated by the sum of the carbohydrate, fat, and protein percentages after multiplying them by the Atwater factors (4, 9, and 4, respectively).

Nutritional assessment of the IP from each settlement, covering school-age children, adults, and the elderly, will then be performed through on-site anthropometric (first year of the project only), biochemical (first year), and dietary (first and second year) assessments. All medical analyses will be conducted by practitioners following established medical protocols to guarantee the welfare and security of participants.

Anthropometric assessments will collect data on body height and weight using a portable stadiometer and a bioelectrical impedance scale. Adult Body Mass Index BMI (kg/m^2^) will be calculated from participants’ measured weight and height, whereas BMI for age will be considered for children aged 5–19 years according to their nutritional status.

Biochemical assessment of venous blood samples taken from each participant after a 12-h overnight fast will be immediately transferred to the hospital laboratory for biochemical analysis. Whole blood samples in EDTA vacutainers will be analysed for complete blood counts using an automated analyser following standard procedures. Other blood samples will be centrifuged and analysed for lipid profiles, including total cholesterol, high-density lipoprotein cholesterol, low-density lipoprotein cholesterol, and triglycerides, using an enzymatic colorimetric technique [46].

Dietary assessments will be collected from an adequate sample size determined according to Eq (1) and divided by demographic group (i.e., school-age children, adults, and the elderly) according to their ratios in the study communities. A 24-hour dietary recall interview will be used to determine individual dietary intake, and food frequency questionnaires will be used to determine food consumption pattern [47]. A repeat 24-h dietary recall will be conducted on a random subset (10%) of the participants on nonconsecutive days from each settlement proportional to the size of each demographic group in order to reduce random measurement error. Dietary assessments will be conducted seasonally (dry and wet seasons) to examine differences in the usual diet between the two seasons. Data will be analysed for energy and nutrient intake using the INMUCAL Nutrients v4 program (INMU, Thailand).

Although project duration and resource constraints do not allow us to explicitly detect or measure nutrition transitions, understood as shifts in traditional Indigenous diets, physical activity, and multiple causes of malnutrition [48], the quantitative and qualitative data collected by our socioeconomic surveys (e.g., wild and marketed foods available to and consumed by the community, expenditure and sources of income by household, roles of local wisdom relevant to the food system, effects of media and information technology on food consumption and lifestyle) and nutritional surveys (e.g., food processing, cooking, consumption practices, nutritional profiles and deficits, and nutrition-related health disorders) will be assessed as indirect evidence of this important issue through comparison with existing previous reports. These include existing reports from education and healthcare agencies as well as previous work by some of our research team members. For example, studies conducted by members of our research team more than a decade ago [30, 31] in one of the Karen communities under study reported indications of community development and change affecting food availability, accessibility, and related concerns by the community leader regarding changes in land and environmental sustainability, culture and tradition, food diversity, and food security. The studies also detected underweight and overweight cases among schoolchildren, raising concerns about diseases associated with nutritional transitions.

### Projecting future changes in traditional food species (WP3)

**Fig 3:**
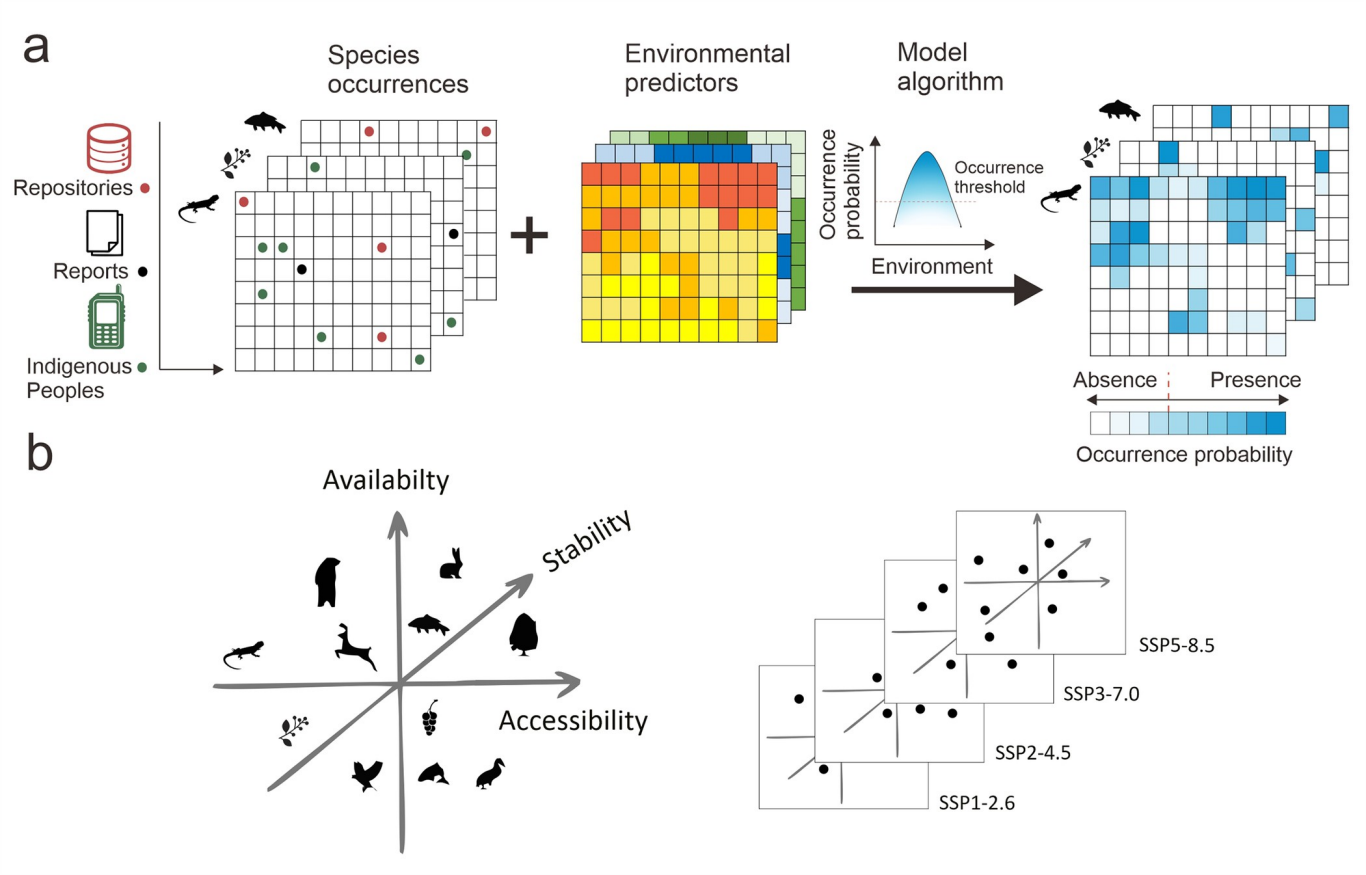
Schematic of the process followed to project future impacts on Indigenous traditional food systems derived from changes in the composition and distribution of wild food species. (a) Species occurrence data collected from a variety of sources (see main text for details), and ecologically-relevant environmental data will be used to develop statistical correlative models (species distribution models) for the prediction of probability of occurrence and range distribution (presence/absence) for the different traditional food species. Models will then be used with different scenarios of climate forcing (RCP) and socioeconomic development (SSP) to provide alternative narratives of future changes in the composition of traditional food systems in terms of their availability (diversity and composition), stability and accessibility to the Indigenous communities.

During the second year of the project, this package will use correlative statistical models (i.e., species distribution models) to project changes in habitat suitability and the range distribution of traditional food plants and animals, representative of each TFS, under a set of alternative future Shared Socioeconomic Pathways (SSPs) and Representative Concentration Pathways (RCPs) with their associated climate change outcomes [49] (Fig 3). These changes will then be translated into projected alterations in the TFS, supporting the studied Indigenous communities (see below). A group of target food species that are important to the community and are representative of the TFS will be selected together with our local Indigenous collaborators, particularly those actively involved in subsistence practices as well as the storage, handling, and preparation of food, and by reference to the information collected by WPs 1 and 2. The importance of a food species will be defined in terms of its nutritional and household contributions, as well as the existence of any other complementary value provided to the community, such as cultural or recreational values.

#### Species occurrence data

Species occurrence data will be collected during the first year of the project from a combination of complementary sources encompassing a range of spatial extents, from regional to local, to adequately capture the environmental correlates driving species distribution (Fig 3). These include (i) public global data repositories (such as Global Biodiversity Information Facility, http://www.gbif.org; iNaturalist, https://www.inaturalist.org; VertNet, http://vertnet.org; or iDigBio, https://www.idigbio.org), (ii) surveys and monitoring programs conducted by regional research and governmental institutions (e.g., the Thungyai Naresuan West Wildlife Sanctuary, within which the two Karen communities are located, and the Institute for Biological Problems of Cryolithozone from the Siberian Branch of the Russian Academy of Sciences), and (iii) data contributed by Indigenous Peoples from the study communities. In the latter case, a small number (4–6 people) of local residents from each community, known as active hunters/collectors, will report the wild food species they collect or hunt during their normal subsistence activities over half a year (i.e., between 2021 and 2022 field visits to capture food seasonality). To do so, they will record the location of the food species using GPS and topographic maps of the area together with the species name and a picture for verification purposes. All collected data will be downloaded into a portable storage device and regularly updated into our project database. Owing to the sensitive nature of the data, the resulting occurrence data collected will only be used for the generation of the models and not disclosed to the public.

The collected occurrence data will follow standard quality check procedures to detect input errors (e.g., duplicate records and invalid coordinates) and perform spatial thinning to remove spatial bias caused by areas with a high density of records in close proximity [50, 51]. To avoid the adverse effects of small sample sizes on model performance, species with fewer than 20 filtered records will not be considered for analysis [52].

#### Environmental predictors

Present and future environmental predictors for modelling the distribution of traditional food species will be sourced from the open-access CHELSA dataset (https://chelsa-climate.org) [53]. CHELSA (Climatologies at high resolution for the Earth’s land surface areas) provides very high resolution (30 arc sec, ~1 km) contemporary and future global climate data based on mechanical statistical downscaling of global reanalysis data or GCM output. The dataset includes a collection of 19 bioclimatic variables derived from summaries (mean conditions, seasonality, and extremes) of monthly temperature and precipitation values, and was developed specifically for species distribution modelling and related ecological applications. Data are available for different periods, including a historical baseline (1981–2010), as well as the present-near future (2011–2040), mid-future (2041–2070) and far future (2071–2100). Variables for future conditions are provided for a number of GCMs and the most up-to-date scenarios from the Coupled Model Intercomparison Project version 6 (CMIP6), which combines different levels of greenhouse gases, radiative forcing (RCPs), and changing socioeconomic factors (SSPs) [53, 54]. Global high-resolution (~5 km) land use data (2015–2100) generated using the Global Change Analysis Model (GCAM) and a land use spatial downscaling model for the RCP-SPP scenarios [55] will also be used to capture the effects of projected anthropogenic land use change in combination with climate change on food species distributions.

The selection of predictor variables for the development of species distribution models (see below) will be made on a case-by-case basis for each food species by selecting those predictors that are deemed to be more relevant, based on the species ecology and advice provided by our local collaborators with traditional knowledge of the species. Collinearity among the selected predictors will then be assessed using pairwise Pearson’s correlation coefficient to avoid highly correlated variables (|*r*| > 0.7) [56].

#### Species distribution models (SDMs)

Development of SDMs for traditional food species will follow recommended best-practices [57–59]. Uncertainty associated with individual modelling algorithms will be minimised by estimating an ensemble of four widely used algorithms: generalised linear models (GLM), generalised additive models (GAM), boosted regression trees (BRT), and maximum entropy (MaxEnt). Following methodological recommendations [60], a sufficiently high (*n* = 10,000) number of pseudo-absences will be randomly selected from locations within our study extent for MaxEnt models, whereas for GLM, GAM, and BRT models, pseudo-absences will be equally balanced against the number of presences. Each of the four model algorithms will be individually tuned for each species to identify associated species-specific optimal predictors [61, 62]. Predictor selection will be based on the optimisation of model parsimony based on the Akaike information criterion for GLM; optimisation of the degree of smoothness using generalised cross validation for GAM [63]; and optimisation of the omission rate and area under the receiver operating curve (AUC) values for a set of candidate models built with different combinations of parameters for BRT and MaxEnt (i.e., learning rate, depth of tree, and bag fraction will be used for BRT [64], and regularisation multiplier and feature class for MaxEnt [65]).

Model performance will be assessed via spatial cross-validation and model accuracy using the AUC and true skill statistic (TSS). Algorithms with poor predictive abilities (AUC < 0.7 and TSS < 0.4) will be excluded from the ensemble [66]. The model predictive capacity will be assessed using the continuous Boyce index [67]. For each species, ensemble predictions of habitat suitability for both contemporary and future periods will then be generated by weighting the predictions from each model algorithm with good predictive ability according to their continuous Boyce index. The continuous habitat suitability projection will be finally binarised to a distribution range (presence/absence) using a 10% presence threshold, allowing us to estimate future changes in the distribution of traditional wild food species under contrasting RCP-SSP scenarios. Projected changes in the distribution and composition of wild food species will then be assessed in terms of expected alterations to the TFS relative to their socioeconomic and nutritional contributions to Indigenous livelihoods. These will be defined in terms of changes in the capacity of TFS to provide sufficiently diverse food (availability) to be harvested locally (accessibility) and obtainable in a reliable manner (stability) to allow IPs to live off the natural environment and maintain traditional practices (Fig 3).

### Estimation of risk (WP4)

Based on the information collected from WPs 1 to 3 and supported by continuous engagement and consultation with representatives of the Indigenous communities and other relevant stakeholders, we will use a narrative-oriented, ‘bottom-up’ approach to risk assessment and guidance for adaptation [68]. This will apply an iterative approach that begins with the semi-quantitative evaluation of the exposure and sensitivity of TFS to projected changes in the composition of food species under alternative emission scenarios. It will then be followed by the qualitative assessment of risks posed by those projected future impacts and the identification of adaptation alternatives.

Classic risk conceptualisation considers risk as the probability of an event relative to the consequences of its occurrence [69]. This relationship can be further decomposed into three elements of risk, namely the hazard, exposure and vulnerability [70]. Whereas hazard represents the probability of occurrence of hazardous events, the combination of exposure and vulnerability (sensitivity and adaptation capacity) refers to the consequences of these hazardous events occurring. Here, we draw on this conceptualisation and adapt it to our study by framing risk as the potential for adverse effects that change the local availability and accessibility of traditional food species generated by future changes in local climatic conditions may have on dependent Indigenous socioecological systems (i.e., the TFS).

Our system is represented by Indigenous communities and the ecosystems with which they interact. The hazard, the impacting force on the asset of interest, comprises future changes in local climate and environmental conditions that translate into changes in the distribution of traditional food species. The asset exposed to the hazard is a traditional food system comprising the pool of food species that are locally available from the environment and are used by the Indigenous community. To capture this dual socioecological dimension of the asset, we develop an index that combines exposure and sensitivity by calculating the level of exposure relative to the degree of human reliance on the asset in terms of its relative contribution to socioeconomic well-being and nutritional security. This approach is conceptually akin to the concept of benefit-relevant indicators [71], which describes how ecosystems provide benefits to society by linking biophysical outcomes to benefits for an identifiable group of people.

Based on an index originally developed for the selection of priority areas in conservation biology [72], our Exposure-Sensitivity (ES) index considers the diversity of locally available food species (i.e., in each grid cell within the study area) and weights the relative importance of each species in that locale by their individual contribution towards Indigenous livelihoods in terms of socioeconomic well-being and nutritional security, giving important species more contribution to the index than those that are less relevant (Table 1).

$$ES=\frac{\sum_{i=1}^{k}\frac{\propto_{i}C_{i}}{n}-1}{\propto_{max}-1}$$ (2)

where *α*_*i*_ is the weight assigned to the *i*th contribution category; *C*_*i*_ is the number of food species present in the local assemblage belonging to the *i*th contribution category; *n* is the total number of species in the local assemblage; *k* is the number of contribution categories represented in the assemblage; and *α*_*max*_ is the weight corresponding to the highest contribution category occurring in the species pool for the entire study area (not just in the local assemblage). The index ranges from 0 when all species belong to the lowest contribution category (accessory socioeconomic and nutritional contributions) to 1 when all species belong to the highest category (critical socioeconomic and nutritional contributions). Hence, this index combines traditional food exposure with the sensitivity of Indigenous communities as a metric of the consequences of climatic hazards that impact the traditional food system. The weights for each contribution category are chosen according to a geometric progression scheme, with the relative contribution of both components (socioeconomic and nutritional) weighted equally (Table 1). Assignment of each food species to a contribution category will be made by consensus through discussions between researchers and IPs based on traditional environmental knowledge of the species and the results from socioeconomic (e.g., relative importance of traditional food to household economies) and nutritional (e.g., proportions of reported food consumption and nutritional/dietary contribution) surveys conducted for WPs 1 and 2.

**Table 1:** The semi-quantitative weight scheme that will be adopted for the computation of the ES index by reference to the individual categories of the food species occurring in each grid cell. Categories refer to the relative contribution of each food species towards Indigenous livelihoods in terms of socioeconomic well-being and nutritional security and their number and criterion will be defined based on expert judgement and the results from the WPs 1 and 2 (see main text).

		Nutritional contribution		
		Accessory	Important	Critical
**Socioeconomic contribution**	**Critical**	*C*_*4*_	*C*_*5*_	*C*_*7*_
		*α*_*4*_ = 8	*α*_*5*_ = 16	*α*_*7*_ = 32
	**Important**	*C*_*2*_	*C*_*3*_	*C*_*5*_
		*α*_*2*_ = 2	*α*_*3*_ = 4	*α*_*5*_ = 16
	**Accessory**	*C*_*1*_	*C*_*2*_	*C*_*4*_
		*α*_*1*_ = 1	*α*_*2*_ = 2	*α*_*4*_ = 8

The projected impacts of climate change on TFS will then be calculated as the relative change in local ES values projected for future periods relative to their current values. The uncertainty associated with these projected impacts will be assessed by comparing the results under different GCMs and alternative emission scenarios to provide a suite of alternative narratives of the nature and degree of impacts that different possible futures may bring.

These alternative narratives will then be presented to representatives of Indigenous communities and other relevant local stakeholders (e.g., the National Park authority) to guide focus-group discussions and qualitatively assess the potential risks posed by projected future impacts of climate change on TFS. Discussions will focus on identifying decision thresholds (i.e., points where the optimal decision changes as a function of risk) based on current adaptive capacity, assessed from local Indigenous knowledge and consideration of the traits of the Indigenous communities that confer resilience to traditional food insecurity, such as economic stability, educational attainment, or job diversity, and the impact scenarios that cross those thresholds.

### Scientific growth, transferability and public outreach (WP5)

The main objective of RISE is to influence policy and contribute to regional development and the long-term sustainability of Indigenous socioecological systems through applied, transferable science. This WP will ensure adequate scientific growth, particularly of our early career team members, meaningful stakeholder engagement, and public outreach throughout the entire lifespan of the project and beyond. The project’s outreach strategy followed a continuous communication strategy. Dissemination of results will be conducted in the form of scientific publications from our case studies and presentations at national and international meetings as well as through the publication of materials such as press-releases, an end-project summary of the main results and conclusions for policy makers. Our project website and social media platforms will be used to disseminate results among, and engage with, the general public. The transferability of results across case studies and elsewhere will be reinforced through the organisation of stakeholder workshops and exploration of international and regional policy implications with a focus on linking our results with the 2030 sustainability agenda and the Paris Agreement on Climate Change. Other relevant forums such as the UN Permanent Forum on Indigenous Issues or the Sustainable Development Working Group of the Arctic Research Council will also be engaged.

**Fig 4:**
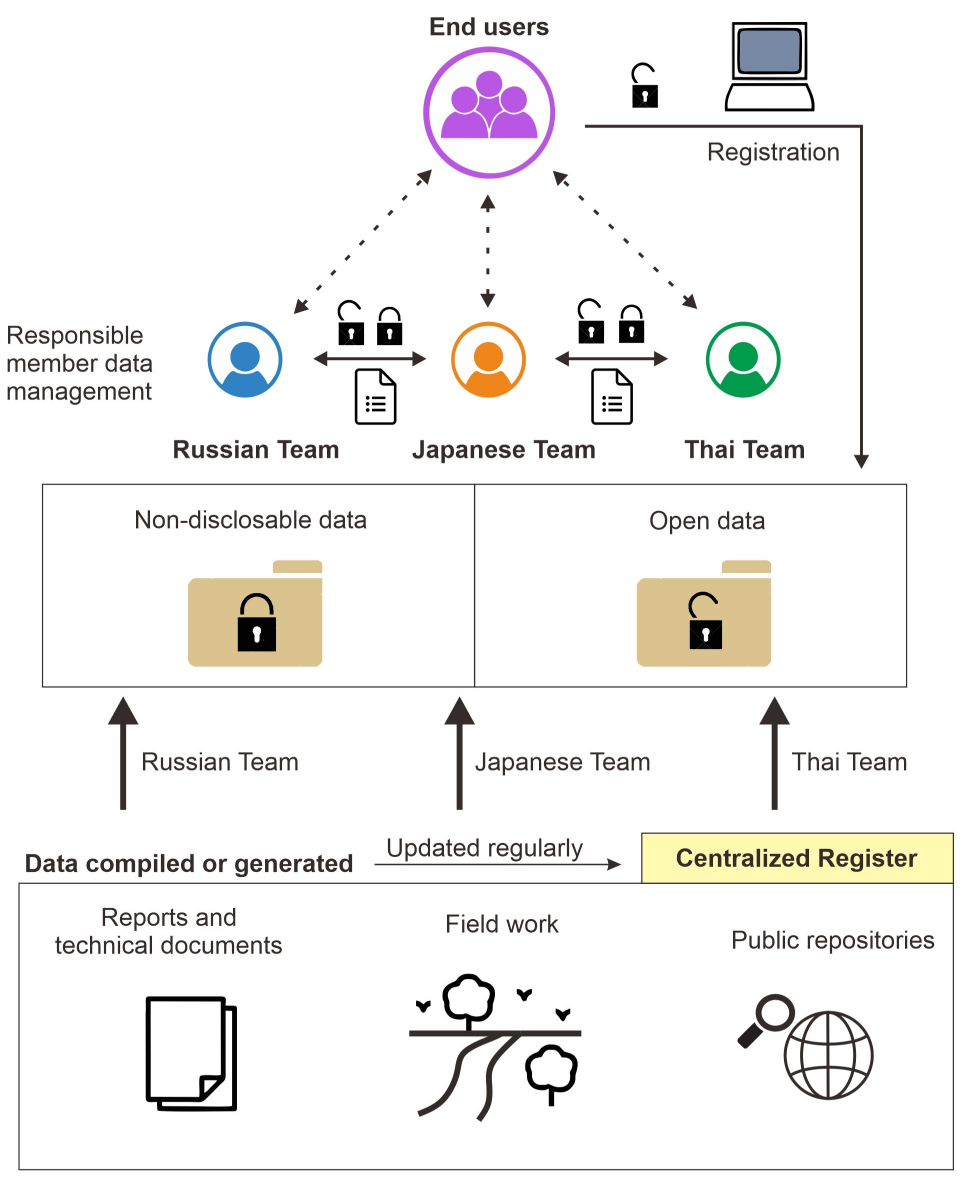
Project data management structure. Data management in RISE will follow a hierarchical structure where each national team is responsible for the documentation, storage and maintenance of all the data generated by them. A designated data manager will act as curator and contact liaison for his/her team data having the main responsibilities of keeping the centralized register up to date and dealing with both internal and external data requests. Internal requests by project team members will be made using a designated request form to keep records of who is using the data and for what purpose. Disclosable data generated by RISE can be consulted through the centralized register available from our project web site and will be made accessible to the public upon registration.

An important deliverable of this work package is the generation of a protocol to define the norms for the use and sharing of data, and the creation of a project database to facilitate both the internal use of data among project members and the efficient release of the open data generated by the project. To do so, RISE will follow a hierarchical structure in which each national team is responsible for the generation, curation, and storage of their own data, as well as to keep an updated centralised register of all data used and generated by the project that project members can internally refer to whenever they need to access data produced by other teams and external users can consult (Fig 4). Each data product listed in the register contains metadata describing its original source, type of data, and status (private or public). This register will be available to the public on our project website and periodically updated and expanded as needed throughout the lifespan of the project.

The data generated by each of the three participating national teams will be handled at the national level by a designated data manager (Fig 4). The data manager will be responsible for the management and storage of the data generated by his/her own team, as well as the registration of that data into the centralised register, and for dealing with data queries of internal or external users regarding the data produced by his/her own team. In principle, all data generated by the project should be internally available to other team members, which is necessary for their research. Specific conditions for data sharing and attribution among team members will be established through joint research agreements between the participating research and funding institutions. As a general rule, project members who want to use data produced by other teams will need to fill in a request form containing details of the data they request and the intended use of the data. Forms will be submitted to the data manager who will process the request by contacting the member of his/her team primarily responsible for that data set and keeping the record of all the request forms submitted to his/her team.

While some of the data we will compile and generate will be copyright or non-disclosable (e.g., sensitive information), other data (particularly processed data produced by our project) will be disclosable and should be made accessible to the public. By making our centralised register public through our project website (currently under construction), end users will be able to check the data produced by RISE’s disclosure status. Disclosable data generated by the project will be made available upon publication of project results and hosted on a public repository accessible upon registration through our website. Registration will require basic user information such as name, email, country, or intended use of data to maintain records of data usage. Users will also be requested to sign an agreement on data-use restrictions for non-commercial purposes.

### Indigenous cultural and intellectual property (ICIP) rights and benefit-sharing

RISE will respect and uphold ICIP rights by following recommended best practice guidelines [32, 33] under the key ethical principles of OCAP (ownership, control, access, and possession) and the Four R’s (respect, relevance, reciprocity, and responsibility) toward those who hold knowledge.

We will follow a principle of ‘active protection’ that promotes the active engagement of researchers with the knowledge holders and relevant community members, ensuring that the data generated by the project is recorded and stored in tangible form and encouraging opportunities for inter‐generational knowledge transmission throughout the project (i.e., intangible knowledge transfer). For example, the local team of collaborators collecting data on the distribution of traditional food species will comprise a mixture of young and experienced hunters/gatherers to facilitate the learning of young people from their elders. RISE is also committed to a ‘two-way’ transfer of knowledge. On one hand, Indigenous People have access to the benefits provided by scientific knowledge and tools. On the other hand, the project integrates and uses Indigenous knowledge (IK) according to existing local customary protocols directing decision making over knowledge sharing, and the free, prior, and informed consent of the Indigenous custodians of that knowledge.

We will follow a continuous process of clear consultation and provide free, prior, and informed consent at the group and individual levels. These will be carried out to ensure that participants in the project have been fully informed and have a clear understanding of the project purpose, methodology, and intended outcomes, including potential uses of the data related to IK generated by the project (i.e., full disclosure of risks and benefits). Participants will be given adequate opportunities and timeframes to make their own decisions regarding their consent to participate without coercion or pressure. Participants will be adequately informed that the consent form is a formal agreement of engagement between the community and the researcher that can nonetheless be revised, suspended, or withdrawn if needed during participation. To ensure correct understanding by all participating members, explanations will be made bilingual, including the official as well as the local Indigenous language, and supported by the use of visuals (time table, diagrams, pictures, information board, etc.) to reinforce the understanding of participants and promote open discussion and communication among the parts. Further, continuous oral and informed consent will be used for those who are illiterate or willing to participate in the project but are reluctant to sign the consent form. More importantly, consent is a necessary protocol that formalises the process of mutual understanding and agreement with the participation of local residents in our project. This is a gradual process that builds on the long-term working relationship of our local teams with the study communities. It will start with the introductory workshops and practical sessions that we are holding in the study communities to provide information on the project rationale, objectives, and operation (approach, materials, milestones, actors, community participation). This will continue throughout the project and beyond, reinforcing co-management and ownership of the project by the Indigenous community through the transparent and open assessment of project outputs, joint decision taking, benefit-sharing, and the discussion of research output with the community and other relevant local stakeholders (e.g., representatives of the Wildlife Sanctuary for the Karen case study).

All information gathered by our project related to IK will be recorded and stored in a form (data products) that is available, and accessible to community members. These include human materials, cultural assets, and knowledge systems, as well as any related products, such as written material (published and unpublished), digital material (e.g., video and audio files, GPS coordinate files), or maps. Data products will be appropriately stored in databases that will protect IK by respecting intellectual property, providing access and benefit-sharing to the IPs, and observing cultural integrity. Any intended external use of data concerning IK will require free, prior, and informed consent from Indigenous participants. Intended uses will first be discussed jointly with the Indigenous participants by presenting in clear, understandable terms the type and format of information/data to be used, specific purpose, dissemination channels (e.g., scientific publication, public lecture, project website, and blog posts), and providing a clear and honest explanation of the potential risks and benefits. We will pay particular attention to ensuring that research reports and project outcomes are not used in any way that is likely to adversely affect the interests of participants and Indigenous communities.

By promoting capacity building through training exchanges and technology transfer to the Indigenous communities, RISE will produce a wide range of direct benefits to the Indigenous community as a whole, as well as the individual members directly collaborating and participating in the project. First, through the active participation and engagement of Indigenous People in the research decision-making process, the project will generate valuable information on potential future risks from climate change to Indigenous traditional food systems with a focus on local needs and Indigenous priorities. This information should help improve the conservation and management of natural resources, as well as open opportunities for regional development and informed discussions with local authorities on the access and use of these natural resources. For example, maps showing projected changes in the future distribution of food species within communal lands, combined with Indigenous traditional environmental knowledge, can provide critical information on potential changes in the future availability and accessibility of food resources, allowing for prioritising management and conservation strategies. This is of special importance where land use and resource accessibility are already restricted, as in the case of the Karen communities and the wildlife sanctuary authority. Similarly, information provided by the project to the community on nutrition (food consumption, growth, and biochemical tests) and the benefits associated with traditional foods (e.g., prevention of non-communicable diseases linked to unhealthy diets and malnutrition) can promote a knowledge-based revitalisation of traditional food use. This is particularly important for the younger generations, because it provides an effective toolkit for adaptation to climate change, making them more resilient through nutritional diversification.

Second, participating members of Indigenous communities will be trained by our research teams on the use of equipment (e.g., GPS) and methodologies for data collection (e.g., how to conduct household interviews, how to operate a GPS, record locations where food species are found, and management of local databases to store data generated). RISE has also provided equipment to support these activities (e.g., GPS devices, solar-powered electricity generators, and digital cameras), which will be donated to the communities once the project runs its course.

Third, by making the data generated by the project accessible and available to participants, RISE will also generate important and valuable information that will help improve their livelihoods. For example, all analytical and anthropometric medical results (including blood analysis) will be shared with the participants and properly explained by a health center/officer, providing further advice and access to medication or intervention if a health problem is reported. This is a rare and important asset for them, as these analyses are expensive and access to health services is often difficult for these isolated communities.

Lastly, Indigenous research collaborators participating in the collection of field data (e.g., household interviews and food species distribution data) will be paid appropriate and agreed upon rates by the project in due recognition of their work.

### Ethics statement

#### Human subject research

The Karen case study (Thailand) has been reviewed by the Mahidol University Central Institutional Review Board (approval number MU-CIRB2021/227.3004; see S1 File).

The Sakha case study (Russian Federation) has been reviewed by the North-Eastern Federal University (NEFU) Biomedical Ethics Committee (approved Resolution No. 6, Report No. 33 on 15 December 2021; see S2 File).

#### Field research

Field permits for the Karen case study are currently being processed by the National Research Council of Thailand (NRCT) and Department of National Parks, Wildlife and Plant Conservation (DNP). Sampling of traditional food species for nutritional analysis will be conducted with permission from the Department of National Parks, Wildlife and Plant Conservation.

The collection of plant and animal material from the field for scientific studies in the Sakha case study is regulated by the Ministry of Ecology, Nature Management, and Forestry of the Sakha Republic (Yakutia). No permit is required for the collection of samples for commonly (not threatened conservation status) available plants, birds, fish, and other animal species. Food samples of licenced species will be purchased from authorised persons and institutions holding official licences for hunting and fishing.

## Discussion

The United Nations 2030 sustainability agenda aims to transform the world for the better by meeting a set of overarching Sustainable Development Goals (SDGs) requiring profound structural changes in society [73] and deep transformations in policy, economy, technology, and science [74]. Important concerns regarding our ability to meet the SDGs remain challenged by biodiversity loss, livelihood inequalities, or climate change impacts on land and ocean systems under the global footprint of extractive and non-regenerative systems [75]. Importantly, the voices, knowledge, and concerns of Indigenous Peoples remain underrepresented in the sustainability agenda [76], despite representing vivid examples of sustainable relationships with nature and equitable distribution of resources among community members. Their voices should be integrated with scientific knowledge and technological advances [77, 78] to implement the restorative and regenerative principles of the much-needed modern circular economies of the future. The sustainability of Indigenous traditional food systems involves not only access to sufficient, safe, and nutritious food harvested from the land, air, and water, but also the preservation of their cultural and religious identity, physical and mental wellbeing, and capacity for self-governance that derives from it. RISE is thus strategically designed not only to address key research questions but also to have a high impact on policymaking and regional development. By linking projections on changes in traditional food systems from alternative future narratives of climate and socioeconomic change to relevant case studies, supported by a solid understanding of the socioeconomic and nutritional dependencies of Indigenous communities on traditional food systems, this project will contribute to improve our understanding of climate change adaptation pathways for ISES. This is key to climate policy and sustainable development. Projections of the future impacts of climate change on TFS, as socioecological systems, are only beginning to be explored, but already point toward important consequences for biocultural relationships [79].

Although we also consider land cover change projections in our species distribution models, our research project focuses mainly on the impacts of climate change on TFS from a socioeconomic and nutritional perspective. However, over and above the impacts of climate change on TFS, the accessibility and availability of (wild and cultivated) traditional foods can be impacted by many other endogenous and exogenous factors. For example, the widespread sociocultural and economic transformations of the Indigenous minority groups in northeast Siberia, resulting from the collectivisation and national assimilation of the Soviet era and reinforced by modern Russian politics, have encroached on their marginalisation from decision-making processes that concern local natural resources and land rights [80, 81]. Similarly, coercing conservationism in the context of Thailand’s globalisation and modernisation saw the emplacement of the Karen communities within the UNESCO World Heritage Site and wildlife sanctuary Thung Yai Naresuan in Thailand as a disruptive factor [82]. The Karen people, who have lived in the area for at least 200 years, have found themselves not only deprived of their right to be consulted and participate in decision making, but also exposed to increasing pressure by the Royal Forest Department and the military to be relocated outside the sanctuary, and to the exhaustive monitoring and management of their activities, leading to longstanding conflicts over biocultural diversity with external actors and institutions who claim control over these areas, invoking superior interests in nature conservation, development, and modernisation [83].

It is also important to note that while we address the socioeconomic and nutritional contribution of traditional food systems to current Indigenous livelihoods, we only account for the future impacts of climate change on livelihoods through its modification of the composition and availability of food species supporting those systems. In other words, we use current socioeconomic and nutritional information to evaluate existing dependencies and contributions, which we use in combination with Indigenous traditional knowledge to assess categories for the exposure-vulnerability index. However, we do not project changes in socioeconomic conditions or possible diet shifts that may result from those changes. This is a very interesting and important topic to cover in future work, but it is outside the scope of our current project, given time and logistic constraints.

Notwithstanding these caveats, we believe that our project represents an important step toward a more holistic and inclusive analysis of the impacts of future climate change on TFS. RISE will promote positive co-management and regional development by providing a forum for the active participation of Indigenous communities in climate change adaptation strategies, with a focus on food-based resources, strengthening the communication between IPs and other governmental resource managers to address and adapt more effectively to climate change impacts. For example, in partnership with local, regional, and state agencies, our project could provide the basis for developing a framework supported by Indigenous knowledge to identify and prioritise the management and conservation of habitats or areas that support culturally important foods and will be most affected by climate change. These types of positive co-management initiatives have demonstrated great success not only in conserving native habitats and traditional food resources but also in safeguarding the right access of IPs to these resources [11].

## Supporting information

S1 Appendix Description of the Indigenous communities that will represent the case studies for the project with indication of the existence of previous socioecological and dietary surveys. (DOCX)

S1 File English translation of the official approval certificate provided by the Mahidol University Central Institutional Board. (PDF)

S2 File English translation of the official approval certificate provided by the North-Eastern Federal University (NEFU) Biomedical Ethics Committee. (PDF)
