# Supplementary material for: Study protocol: International joint research project ‘climate change resilience of Indigenous socioecological systemsʼ (RISE)
Source: PLoS One. 2022 Jul 21;17(7):e0271792. doi: 10.1371/journal.pone.0271792 (PMC9302735; doi:10.1371/journal.pone.0271792)
Supplement: S1 Appendix — (DOCX) [file pone.0271792.s001.docx]

**Table S1**. Description of the indigenous communities that will represent the case studies for the project with indication of the existence of previous socioecological and dietary surveys. The 18 settlements listed for the Sakha Republic will be subject to a general socioeconomic survey during the first year of the project, then two settlements will be selected from the second year to conduct a systematic all-household survey and the nutritional analyses according to those conducted for the Karen case study in Thailand (see Material and Methods for details). Accordingly, sample sizes for the Russian settlements are based on the distribution of the total sample size (*n* = 400 respondents) among each of the 5 economic zones in Yakutia (i.e., Arctic and southern, central, eastern and western Yakutia), according to class sizes of population in working age and older for each rural settlement (≤500, 501-1,000, 1,001-2000, ≥2,001 people corresponding to 10-15, 20-25, 30 and 35 respondents respectively). The total sample size was first calculated based on the total (i.e., across all 18 settlements) population in working age and over (*N* = 12,231) using the Slavin formula for a precision level of 0.05. The population column provides the total population by settlement together with the population in working age and older between brackets. For the Karen case study, the socioeconomic survey consist of an all-household approach. Sample sizes in this case are instead provided for the nutritional assessment and were also calculated using the Slavin formula (0.05 precision) on the population of each settlement in school age (6-12 years) and over provided between brackets in the population column. Total population figures in the population column refer to the most recent estimates available. Because the last official population census in Sakha Republic took place in 2010 (next census scheduled for the fall of 2021 with data expected to be available in 2022), we provide instead estimates made by the Russian Federal State Statistic Service (https://rosstat.gov.ru/compendium/document/13282). The population data of the Thai team is provided by The Bureau of Registration Administration under the Department of Provincial Administration (https://stat.bora.dopa.go.th/stat/statnew/statMONTH/statmonth/#/mainpage).

| **Settlement** | **District** | **Ethnic group** | **Traditional activities** | **Sociological survey** | **Dietary survey** | **Population** | **Sample size** |
| --- | --- | --- | --- | --- | --- | --- | --- |
| **SAKHA REPUBLIC CASE STUDY (Russia)** | | | | | | | |
| Pokhodsk | Nizhnekolymsky | Russian old-timers, Chukchi, Ukagirs | Tundra reindeer husbandry, fishing |  | 2013- | 266 (201) | 10 |
| Kharyallakh | Oleneksky | Evenks | Forest-tundra reindeer husbandry, hunting, gathering |  |  | 855 (646) | 25 |
| Bykov Mys | Bulunsky | Russian old-timers, Evenks, Evens, Ukagirs | Fishing, Tundra reindeer husbandry, hunting |  |  | 514 (388) | 15 |
| Chokurdakh | Allaikhovsky | Russian old-timers, Ukagirs | Fishing, Tundra reindeer husbandry |  |  | 2077 (1568) | 35 |
| Russkoe Ystye | Allaikhovsky | Russian old-timers, Ukagirs | Fishing, Tundra reindeer husbandry |  |  | 127 (96) | 10 |
| Ugoyan | Aldansky | Evenks | Taiga reindeer husbandry, hunting | 2017-2019 | 2013- | 375 (283) | 10 |
| Betuntsy | Amginsky | Sakha | Cattle breeding, gathering | 2017-2019 | 2013- | 1209 (913) | 30 |
| Isit | Khangalassky | Sakha | Cattle and horse breeding, hunting, gathering |  |  | 272 (205) | 10 |
| Ulakhan-An | Khangalassky | Sakha | Cattle and horse breeding, hunting, gathering |  | 2013- | 1133 (855) | 30 |
| Kobyay | Kobyaysky | Sakha | Fishing, hunting |  |  | 2289 (1728) | 35 |
| Magaras | Gorny | Sakha | Cattle and horse breeding, hunting, gathering |  | 2013- | 1044 (788) | 25 |
| Edeytsy | Namsky | Sakha | Cattle and horse breeding, hunting, gathering | 2017-2019 | 2013- | 1202 (908) | 30 |
| Rassoloda | Megino-Kangalassy | Sakha | Cattle and horse breeding, hunting, gathering |  |  | 431 (325) | 15 |
| Kharbala | Verkhnevilyuysky | Sakha | Cattle and horse breeding, hunting, gathering | 2017-2019 | 2013- | 593 (448) | 20 |
| Lekechen | Viluisky | Sakha | Cattle and horse breeding, hunting, gathering |  |  | 441 (333) | 15 |
| Chappanda | Nurbinsky | Sakha | Cattle and horse breeding, hunting, gathering |  |  | 797 (602) | 25 |
| Elgey | Suntarsky | Sakha | Cattle and horse breeding, hunting, gathering |  | 2013- | 1660 (1253) | 35 |
| Topolinoe | Tomponsky | Evens | Mountain taiga reindeer husbandry, hunting |  |  | 915 (691) | 25 |
| **KAREN CASE STUDY (Thailand)** | | | | | | | |
| Sanephong | Sangkhlaburi | Karen | Fallow period, hunting, gathering, fishing, farming | 2005-2007 and 2010-2012 | 2005-2007 and 2010-2012 | 1157 (696) | 254 |
| Kao-Sa-Deng | Sangkhlaburi | Karen | Fallow period, hunting, gathering, fishing, farming | 2010-2012 | 2010-2012 | 361 (262) | 158 |
