# Supplementary material for: Study protocol: International joint research project ‘climate change resilience of Indigenous socioecological systemsʼ (RISE)
Source: PLoS One. 2022 Jul 21;17(7):e0271792. doi: 10.1371/journal.pone.0271792 (PMC9302735; doi:10.1371/journal.pone.0271792)
Supplement: S1 File — (PDF) [file pone.0271792.s002.pdf]

COA No. MU-CIRB 2021/163.1207

Mahidol University Central Institutional Review Board

Certificate of Approval

Protocol No.: MU-CIRB 2021/227.3004

Title of Project: Climate change Resilience of Indigenous SocioEcological systems (RISE)

Type of Review: Expedited Review

Approval Includes:

1) Principal Investigator: Assoc. Prof. Wantanee Kriengsinyos

   Affiliation: Institute of Nutrition, Mahidol University

   Research Site: Institute of Nutrition, Mahidol University
2) Submission Form Version Date 7 July 2021
3) Protocol Version Date 7 July 2021
4) Participant Information Sheet Version Date 6 July 2021
5) Information Sheet for Legal Guardian – Participants Age 0-6 Years Version Date 6 July 2021
6) Information Sheet for Legal Guardian – Participants Age 7-12 Years Version Date 6 July 2021
7) Assent Form for Children 7-12 Years Version Date 29 April 2021
8) Informed consent form for legal guardian – participants age 0-6 years Version Date 29 April 2021
9) Informed consent form for legal guardian – participants age 7- 12 years Version Date 29 April 2021
10) Informed consent form for research participants (age 18 years or older) Version Date 29 April 2021
11) Questionnaire Version Date 31 June 2021
12) Case Record Form Version Date 31 June 2021

MU-CIRB is in Full Compliance with International Guidelines for Human Research Protection such as Declaration of Helsinki, The Belmont Report, CIOMS Guidelines and the International Conference on Harmonization in Good Clinical Practice (ICH-GCP)

Date of Approval: 12 July 2021

Date of Expiration: 11 July 2022

Signature of Chairperson: ....

(Emeritus Professor Dr. Wariya Chinwanno)

MU-CIRB Chair

* See *list of Co-Investigators at the back page*

List of Co – Investigators

1. Dr. Pattamaporn Joompa
2. Assoc. Prof. Solot Sirisai
3. Dr. Kritsada Bunchai
4. Ms. Sinee Chotiboriboon
5. Dr. Sueppong Gowachirapant
6. Ms. Prapa Kongpunya
7. Ms. Nattapach Thongkam
8. Ms. Nutcharee Setapan

All MU-CIRB Approved Investigators must comply with the Following:

1. Conduct the research according to the approved protocol.
2. Conduct the informed consent process without coercion or undue influence, and provide the potential subjects sufficient time to consider whether or not to participate.
3. Use only the Consent Form bearing the MU-CIRB Approval stamp.
4. Obtain approval of any changes in research activity before commencing and informed research participants about the changes for their consideration in pursuing the research.
5. Timely report of serious adverse events to MU-CIRB and any new information that may adversely affect the safety of the subjects or the conduct of the trial.
6. Provide MU-CIRB the progress reports at least annually or as requested.
7. Provide MU-CIRB the final reports when completed the study procedures.

....

MU-CIRB Address: Office of the President, Mahidol University, 4th Floor, Room Number 411
999 Phuttamonthon 4 Road, Salaya, Nakhonpathom 73170, Thailand
***Tel:** 66 (0) 2849 6224, 6225 **Fax:** 66 (0) 2849 6224*
***E-mail:** mucirb@gmail.com*
***Website:** http://www.sp.mahidol.ac.th*
