# Supplementary material for: Study protocol: International joint research project ‘climate change resilience of Indigenous socioecological systemsʼ (RISE)
Source: PLoS One. 2022 Jul 21;17(7):e0271792. doi: 10.1371/journal.pone.0271792 (PMC9302735; doi:10.1371/journal.pone.0271792)
Supplement: S2 File — (PDF) [file pone.0271792.s003.pdf]

Ministry of Science and Higher Education of the Russian Federation
Federal State Autonomous Educational Institution of Higher Education «M. K. Ammosov North-Eastern Federal University»
58 Belinsky str. Yakutsk, 677000
The Republic of Sakha (Yakutia)
Tel. (4112) 35-20-90
Fax (4112) 32-13-14
E-mail: rector-svfu@ysu.ru
http://www.s-vfu.ru

_______________№___99____________

to №__99________dated_ 01.12.2021________

Local Biomedical Ethics Committee

CERTIFICATE OF APPROVAL
Resolution No. 6
Report No. 33, December 15, 2021

Attendees: MD, Professor N.V. Savvina; MD O.V. Tatarinova; MD, Professor G.A. Palshin; MD., Candidate, Associate Professor M.N. Petrova; chief nurse of the Medical Center of Yakutsk A.V. Egorova.

Research project title: Climate Change Resilience of Indigenous SocioEcological Systems (RISE).
Research timeframes: January 2022 – December 2023.
Type of research: the Russian Foundation for Basic Research project No. 21-55-70104.
Discipline: Interdisciplinary.

The following documents were submitted for consideration:
1. Agreement between the Russian Foundation for Basic Research and NEFU No. 21-55-70104\21 on entitling a grant to the winner of the competition and implementing a research project.
2. Research summary.
3. List of criteria for inclusion or non-inclusion of respondents in the research and exclusion of respondents from the research.
4. Research plan (structure, design).
5. Key parameters of the research method.
6. Information for the respondents with the Informed Consent Forms.
7. Confidentiality commitment.
8. List of clinical research centers.
9. A respondent's Case Report Form.
10. Questionnaire for the socio-economic survey of indigenous peoples in the Sakha Republic (Yakutia).
11. 24-hour dietary recall form.
12. Curriculum vitae (CV) of the Principal Investigator Tuyara Gavrilyeva.

Having considered the submitted documents, the Ethics Committee decided as follows:
1. The research project aims to study current and project future risks from climate change and socioeconomic development to the natural capital supporting traditional indigenous socio-ecological systems (ISS). Two distinctive ISS (Thailand and Siberia) will be used as case studies. RISE will deliver high impact science to inform effective decision making towards regional development, sustainability, and climate change adaptation. Hence, the goal and objective of the project is to ensure availability of medical care to the population, thus allowing to decrease morbidity and mortality from chronic non-infectious and infectious diseases, improve the quality of medical care for treatment thereof, and help reduce the cost of treatment and rehabilitation of respondents (patients).
2. The research fully complies with ethical standards and legal rules. Members of the Ethics Committee approve the research project, including: 2.1. Doctor of Economics, Research Professor at NEFU Institute of Engineering & Technology Tuyara GAVRILYEVA as the

Principal Investigator;
2.2. Work Packages and Research Methods of the project submitted for expert review:

- WP 1. Socioeconomic analysis of ISS, including systematic household surveys based on the questionnaire of socio-economic survey of indigenous peoples in the SR(Y), extended dietary surveys and focus groups, collection of data on traditional diet and other information.
- WP 2. Nutritional/dietary analysis of ISS, including food consumption patterns of various socio-demographic groups of indigenous peoples of the SR(Y) based on a special questionnaire; actual nutrition based on full-day (24-hour) diet study using a special form (24-hour dietary recall form); collection of anthropometric data using a stadiometer and smart scales to calculate body mass indexes (BMI); collection of venous blood samples (general blood count (hemoglobin, hematocrit, erythrocytes, leukocytes, platelets), blood biochemistry and lipid profile (total cholesterol,
triglycerides, LDL Cholesterol, HDL Cholesterol, blood glucose measurement); blood pressure; analysis and statistical processing of the data obtained.
2.3. Research plan (structure, design) for 2022-2023: field research will be undertaken in two settlements of the Sakha Republic (Yakutia), selected for a comprehensive study based on the first stage of field work in 2021. As part of WP 1 and 2, at least 100 respondents will be randomly selected in each of the settlements. The total number of respondents in two settlements will make at least 200 people. Study groups: '6-12 years' (only collection of venous blood and anthropometric data, blood pressure measurement), '19-59 years' and '60 years and older'.
2.4. Informed Consent Forms of a research participant (respondent);
2.5. Information for the respondents;
2.6. A respondent's Case Report Form;
2.7. Socio-economic survey questionnaire;
2.8. 24-hour dietary recall form.
2.9. Clinical research centers:
- Clinic of the Institute of Medicine, M.K. Ammosov NEFU, 36 Kulakovskogo St, Yakutsk, Russian Federation, Tel.: 8-800-100-14-03.
- The Institute of Nutrition of Mahidol University (INMU), 999 Phutthamonthon 4 Rd., Salaya, Phutthamonthon, Nakhon Pathom 73170, Thailand, Tel.: 0-2800-2380 Fax.: 0-2441-9344.

2.10. Members of the research team involved in the project field work:
- Nadezhda MAKSIMOVA, Doctor of Medical Sciences, Head of the Molecular Medicine and Human Genetics Research Laboratory, NEFU Institute of Medicine, Professor of the Department of Neurology and Psychiatry, NEFU Institute of Medicine;
- Leonid ZHOZHIKOV, Doctoral student, Department of Neurology and Psychiatry, NEFU Institute of Medicine;
- Varvara PARILOVA, Doctoral student, Department of Sociology and Human Resources Management, NEFU Institute of Finances and Economics;
- Vyacheslav GABYSHEV, Head of the Department of Science, Environmental Education, and Procurement of 'Bear Islands' State Nature Reserve.

The approved investigators shall comply with the following requirements:
1) Undertake the research in compliance with the approved protocol;
2) Conduct the informed consent process without coercion or undue influence and provide potential subjects sufficient time to consider whether or not to participate in the study;
3) Use only approved questionnaires and forms;
4) Obtain approval of any changes to research activities before commencing and inform study participants about such changes for their consideration in pursuing the research;
5) Provide the Ethics Committee with documents for follow-up monitoring (Appendix 11 on the Committee's page at the NEFU website (https://www.s-vfu.ru/universitet/rukovodstvo-i-

struktura/instituty/mi/nauchnaya- worka/%D0%9A%D0%AD/).

6) Provide the Ethics Committee with documents for reviewing reports of adverse events (Appendix 12 on the Committee's page at the NEFU website).

7) Timely inform the Ethics Committee about inconsistencies/violations of the protocol or the closure of the research protocol using a special form (Appendices 13 and 14 on the Committee's page at the NEFU website).

8) Submit to the Ethics Committee a report on the research undertaken after completion of the protocol as per the special form within 15 working days after the expiration of this approval (Appendix 15 on the Committee's page at the NEFU website).

The Local Biomedical Ethics Committee operates in accordance with the Regulation approved by the quality management system (QMS-P-1.8-163-13, Version 1.0, 2014). In its activities, the Ethics Committee is guided by the legislative and regulatory acts of the Russian Federation, as well as by international ethical principles and rules for conducting scientific research involving humans or laboratory animals, set out in internal, national, and international documents, including: the Charter of the NEFU, the Constitution of the Russian Federation, the Federal Law 'On fundamentals of protecting the health of citizens of the Russian Federation' (No. 323-FZ dated November 21, 2011), the Order of the State Commission for Academic Degrees and Titles 'On the procedure for conducting biomedical research in humans' (published in the SCADT Bulletin 2002, No. 3), the 1964 World Medical Association's Declaration of Helsinki (as supplemented in 1975, 1983, 1989, 1996, 2000, 2002, 2004); guidelines and recommendations of the UNESCO Ethics Office, World Health Organization, European Forum for Good Clinical Practice, Council for International Organizations of Medical Sciences; European Convention for the Protection of Vertebrate Animals used for Experimental and other Scientific Purposes (Strasbourg, March 18, 1986, ETS No. 123), and others.

Ethics Committee Chair
Doctor of Medical Sciences,
Professor N.B. Savvina

Ethics Committee Secretary,
Candidate of Medical Sciences,
Associate Professor M.N. Petrova

Date of Approval: December 15, 202[illegible]
Date of Expiration: December 31, 2023

ЗАВЕРЯЮ
Начальник УРПиКП СВФУ
Тимофеева Л.М.
«__» мая 2022 г.
